# Supplementary figures and images for: You are what you eat: fungal metabolites and host plant affect the susceptibility of diamondback moth to entomopathogenic fungi
Source: PeerJ. 2022 Dec 19;10:e14491. doi: 10.7717/peerj.14491 (PMC9774005; doi:10.7717/peerj.14491)

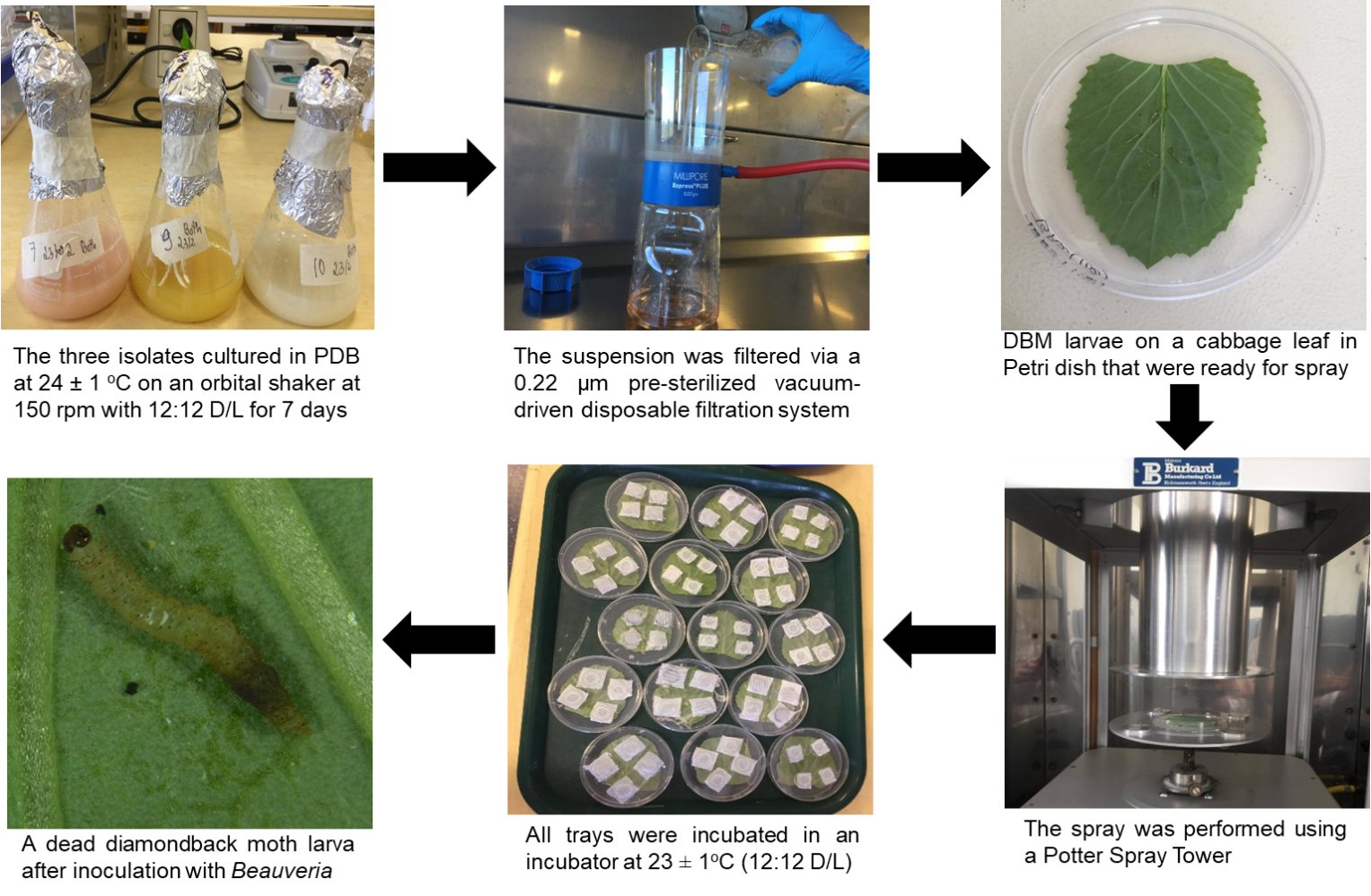

Supplement: Supplemental Information 1 [file peerj-10-14491-s001.png]
